# Adherence to the guidelines and the pathological diagnosis of high-risk gastrointestinal stromal tumors in the real world

**DOI:** 10.1007/s10120-019-00966-4

**Published:** 2019-04-30

**Authors:** Toshirou Nishida, Yoshiharu Sakai, Masakazu Takagi, Masato Ozaka, Yuko Kitagawa, Yukinori Kurokawa, Toru Masuzawa, Yoichi Naito, Tatsuo Kagimura, Seiichi Hirota, The members of the STAR ReGISTry Study Group, Takuro Saito, Yoshito Komatsu, Masato Kondo, Tsutomu Hayashi, Yoshiharu Sakai, Naoto Gotoda, Nobuhiro Takiguchi, Atsuhiko Maki, Hideo Baba, Hajime Orita, Hiroshi Yabusaki, Gaku Chiguchi, Dai Manaka, Kazuhito Nabeshima, Hiromitsu Akabane, Koichi Ono, Norihito Wada, Masahide Kaji, Kazuhiro Yoshida, Ikuo Takahashi, Kazumasa Fujitani, Sohei Matsumoto, Yutaka Tamamori, Hiroaki Saito, Shugo Ueda, Masahiro Yamamura, Hirofumi Fujii, Shigefumi Yoshino, Akihiro Suzuki, Eigo Otsuji, Shigeyuki Kawachi, Tsuyoshi Takahashi, Kazuya Muguruma, Suguru Ishikawa, Masaaki Mitsutsuji, Hiroshi Takamori, Takashi Kaiho, Akihiro Sako, Seiji Ito, Masahiro Mori, Makoto Tokuhara, Yoshihiko Kawaguchi, Naoki Hirabayashi, Motohira Yoshida, Masazumi Takahashi, Shiro Takase, Keishi Yamashita, Yoshiaki Iwasaki, Yutaka Ozeki, Yasunori Nishida, Keisuke Koeda, Toshimasa Tsujinaka, Hiroshi Kanie, Shinji Hato, Junya Morimoto, Hiroshi Honda, Hirotaka Tashiro, Yoshihiro Kakeji, Hiroaki Hata, Toshiro Sugiyama, Takayuki Nobuoka, Ryoji Fukushima, Katsuro Sugiyama, Junichi Hasegawa, Tsunehiro Yoshimura, Atsuo Takashima, Chikara Kunisaki, Hiroharu Shinozaki, Naoto Senmaru, Hiroshi Imamura, Satoshi Otsu, Daisuke Kobayashi, Akinori Noguchi, Akinori Takagane, Atsushi Mitsunaga, Shigeyuki Tamura, Jin Matsuyama, Yoshio Oka, Kiyoshi Kajiyama, Takuji Yamada, Sumito Hoshino, Hideki Ohdan, Tomokazu Kakishita, Katsuhiko Yanaga, Yasushi Rino, Takayuki Takahashi, Hisahiro Matsubara, Masahiro Ishizaki, Songtae Kim, Noriyuki Inaki, Noriyuki Hirahara, Masaru Morita, Tatsuo Kanda, Tomoki Yamatsuji, Mitsutoshi Tatsumi, Chikara Ebisui, Yoshifumi Ikeda, Tsukasa Inoue, Wataru Kimura, Hiroyuki Nakaba, Takamune Shibaji, Taichi Tatsubayashi, Masahiro Sakon, Yo Isobe, Mitsuo Shimada, Mitsuru Sasako, Hirofumi Tomori, Koichi Demura, Masahiro Fujikawa, Hirohito Ishizuka, Masanari Tendo, Shinichi Sakuramoto, Akiyoshi Kanazawa, Norimasa Fukushima, Seiji Sato, Takaomi Takahata, Tetsuya Kusumoto, Takeshi Omori, Masanobu Takahashi, Masahiro Inoue, Norimitsu Tanaka, Motoki Ninomiya, Yasufumi Teramura

**Affiliations:** 1 Department of Surgery, National Cancer Center Hospital, 5-1-1 Tsukiji, Chuoku, Tokyo, 104-0045 Japan; 2 Department of Surgery, Kyoto University Hospital, Kyoto, Japan; 3 Department of Gastroenterological Surgery, Shizuoka General Hospital, Shizuoka, Japan; 4 Department of Gastroenterological Medicine, Cancer Institute Hospital Japanese Foundation for Cancer Research, Tokyo, Japan; 5 Department of Surgery, Keio University Hospital, Tokyo, Japan; 6 Department of Gastroenterological Surgery, Osaka University Graduate School of Medicine, Suita, Japan; 7 Department of Surgery, Osaka Police Hospital, Osaka, Japan; 8 Department of Breast and Medical Oncology, National Cancer Center Hospital East, Kashiwa, Japan; 9 Translational Research Center for Medical Innovation, Foundation for Biomedical Research and Innovation at Kobe, Kobe, Japan; 10 Department of Surgical Pathology, Hyogo College of Medicine, Nishinomiya, Japan; 11 Present Address: Department of Surgery, Kansai Rosai Hospital, Amagasaki, Japan

**Keywords:** Gastrointestinal stromal tumor, Guidelines, Pathological diagnosis, Multidisciplinary board, Adjuvant therapy

## Abstract

**Background:**

A multidisciplinary approach based on guidelines and pathological diagnosis by specialized pathologists are important for improving the prognosis and QoL of GIST patients. This study examined the adherence to the guidelines and the concordance of the pathological diagnosis of high-risk GISTs.

**Patients and methods:**

Among 541 patients with high-risk GISTs recruited to the prospective registry between Dec. 2012 and Dec. 2015, 534 patients were analyzed after central pathology with KIT and DOG1 IHC and genotyping of *KIT* and *PDGFRA*.

**Results:**

Of the 534 patients, 432 (81%) received imatinib adjuvant therapy at a starting dose of 400 or 300 mg/day. Multivariate analysis indicated that age (HR 0.71; 95% CI 0.58–0.88), tumor size (HR for > 10 cm vs < 5 cm, 3.87; 95% CI 1.72–8.74), mitosis (HR for > 10 vs < 5, 3.54; 95% CI 1.84–6.79), tumor rupture (HR 3.69; 95% CI 1.43–9.52) and performance status (HR 0.55; 95% CI 0.31–0.99) were independently related to adjuvant therapy. Among the 534 high-risk GISTs diagnosed locally, 19 tumors (3.6%) were diagnosed as non-GISTs, and the other 93 (18.1%) GISTs were reclassified into lower risk categories by central pathology. Among 10 patients with non-GISTs and 8 patients with *PDGFRA* D842V mutations, 4 (40%) and 3 (38%) patients, respectively, continued the therapy after receiving the central pathology results.

**Conclusions:**

The adherence to guidelines and the concordance of pathological diagnoses were comparatively good for high-risk GISTs. Central pathology may contribute to improved diagnosis, but further refinements may be required.

**Electronic supplementary material:**

The online version of this article (10.1007/s10120-019-00966-4) contains supplementary material, which is available to authorized users.

## Introduction

Rare cancers present several challenges, including delays in the diagnosis and the administration of treatments that are not always based on the guidelines used in clinical practice [1]. Furthermore, there is significant discordance in the pathological diagnosis of rare cancers between disease-specific pathologists and general pathologists, which rarely occurs for common cancers [2]. Soft tissue sarcoma is a typical rare cancer with a wide variety of histological subtypes originating from connective tissue, bone, and viscera. In sarcoma, major discordance rates of 10–20% in the pathological diagnosis between disease-specific and general pathologists has been reported, and a rate of minor pathological discrepancies, such as different grading, of 20% has been reported [3-5]. Gastrointestinal stromal tumors (GISTs) are soft tissue sarcomas with established diagnostic criteria based on KIT and DOG1 immunostaining and genotyping [6-8]. The established treatment for GISTs is imatinib, sunitinib or regorafenib after the discovery of driver mutations in the *KIT* or *PDGFRA* gene [9]. These facts may indicate that GISTs might differ from other soft tissue sarcomas in terms of the pathological diagnosis and in clinical practice; however, the concordance of the pathological diagnosis of GISTs in the real world is still unknown, and factors related to this pathological discordance have yet to be investigated.

Diagnosis and treatment based on guidelines and sharing the treatment decision with patients under the supervision of a multidisciplinary team are suggested to improve the quality of life (QoL) and prognosis of patients [10]. The first clinical practice guidelines for GISTs were published by the National Comprehensive Cancer Network (NCCN) in 2004 [6], followed by guidelines published by the European Society for Medical Oncology (ESMO) [7], and they have been revised along with the development of innovative medicine. With an increase in the amount of established clinical evidence, each country, including Japan [8], has its own clinical practice guidelines for GISTs. Although many GIST guidelines have been published and revised several times, there are few reports on the clinical use and adherence to the guidelines in clinical practice [11-13]. We prospectively registered patients with high-risk GISTs diagnosed in local hospitals and institutions. Using data obtained from the registry study, we examined the concordance of pathological diagnoses, measured the discordance between the guidelines and clinical practice in terms of adjuvant therapy in the treatment of high-risk GISTs, and investigated potential causes of the discordance.

## Patients and methods

### Study design

Between Dec. 2012 and Dec. 2015, a total of 541 patients with high-risk GISTs who were pathologically diagnosed and treated in each participating hospital were recruited to participate in the prospective observational registry study (STAR ReGISTry) (Supplemental Fig. 1). The modified National Institutes of Health (NIH) consensus criteria were used for risk stratification in this study [14]. As two patients were later revealed to have intermediate-risk GISTs by the local diagnosis, one tumor was a recurrent tumor, and one registration was a duplicate, a total of 537 patients were eligible for registration. In our STAR ReGISTry, we also collected surgical specimens for the central pathology and genotyping of the *KIT* and *PDGFRA* genes conducted by one of the authors. In the central pathology, a disease-specific pathologist reviewed all materials and data of hematoxylin and eosin staining, immunohistochemistry staining, and genotyping using obtained surgical specimens. Among the 537 patients, central pathology and genotyping findings were available for 534 patients, as three patients lacked specimens (Supplemental Fig. 1). Ethical approval for the study was initially obtained from the institutional review board (IRB) of the National Cancer Center and then from those of the other participating hospitals. Signed informed consent was obtained from all participating patients. This trial is registered with the UMIN Clinical Trials Registry, number UMIN000009531.

In the central pathological examinations, hematoxylin and eosin (H&E) staining and immunohistochemistry (IHC) for KIT, DOG1, CD34 and Ki67 were performed as previously described [9, 15]. The Ki-67 labeling index was calculated as a percentage of Ki-67-positive tumor cells to all tumor cells in approximately 1000 cells of the hot spot. Since the field number of the microscope used in the central review was 26.5, 50 high-power fields (HPFs) corresponded to 17.235 mm^2^. Genotyping was performed after the extraction of genomic DNA from the paraffin sections using the QIAamp DNA Mini Kit (QIAGEN, Hilden, Germany); exons 9, 11, 13, and 17 of the *KIT* gene and exons 12, 14, and 18 of the *PDGFRA* gene were amplified by PCR and sequenced as described elsewhere [9, 15].

In this research, we also conducted a questionnaire survey in each participating hospital as research accompanying the STAR ReGISTry. The study was approved by the IRB of the Osaka Police Hospital and the National Cancer Center. These studies were conducted in accordance with the World Medical Association Declaration of Helsinki, Ethical Principles for Medical Research Involving Human Subjects (Amended in Seoul in October 2008) and the Ethics Guidelines for Clinical Research (Ministry of Health, Labour and Welfare Notice no. 415, 2008).

### Statistical analysis

The demographics and baseline characteristics of the patients with and without adjuvant therapy were compared, and differences were tested using the Chi-squared test or the Mann–Whitney *U* test to identify risk factors associated with non-adherence. Multiple logistic regression analysis of the demographics and baseline characteristics as covariates was performed, and variable selection was performed using a stepwise method. The data were analyzed using SAS Version 9.4 (SAS Institute, Inc., Cary, NC, USA). This report is based on baseline data fixed on January 31, 2018.

## Results

The enrolled patients included 294 (55%) men and 240 (45%) women, with a median age of 65 years. GISTs were located in the stomach (*N* = 318; 60%), small intestine (163; 31%), colon and rectum (32; 6%), esophagus (7; 1%) and extragastrointestinal tissues (14; 3%) (Table 1). The median tumor size was 7.5 cm, and the median mitotic count was 10/50 HPFs, as determined by the diagnosis at local hospitals. Tumor rupture, including 33 preoperative ruptures and 29 intraoperative ruptures, was observed in 66 (12%) patients. Histologically, the tumors comprised 446 (84%) spindle cell-type tumors, 17 (3%) epithelioid cell-type tumors and 46 (9%) mixed cell-type tumors. Open surgery was performed in 387 (72%) cases, and laparoscopic surgery was performed in 147 (28%) cases. Clinically significant morbidities due to surgery were observed in 63 patients, and most were less than grade 2 (Supplemental Table 1). No deaths related to surgery occurred.

**Table 1:** Patients characteristics

Total	(*N* = 534)
Age (median, IQR; years)	65 (56–72)
Gender	
Male	294 (55%)
Female	240 (45%)
PS	
0	447 (84%)
1	76 (14%)
2	4 (1%)
3	3 (1%)
Unavailable	4 (1%)
Location	
Esophagus	7 (1%)
Stomach	318 (60%)
Small intestine	163 (31%)
Colon and rectum	32 (6%)
Others	14 (3%)
Neoadjuvant therapy	
(−)	475 (89%)
(+)	59 (11%)
Surgery	
Open	387 (72%)
Laparoscopic	147 (28%)
Curability of surgery	
R0	517 (97%)
R1	17 (3%)
Tumor size (cm; *n* = 533)	7.5 (5.5–11.3) (median, IQR)
Unknown	1
Mitosis (/50HPF; *n* = 497) at local 10 (5–23) (median, IQR)	
Unknown	38
Tumor rupture	
No	459 (86%)
Yes	66 (12%)
Preoperative	33 (6%)
Intraoperative	29 (5%)
Unknown	9 (2%)
Histological types	
Spindle	446 (84%)
Epithelioid	17 (3%)
Mixed	46 (9%)
Unavailable	25 (5%)
Genotyping	
KIT	457 (86%)
PDGFRA	18 (3%)
Wild type	36 (7%)
Unavailable	22 (4%)

Of 534 patients, 432 (81%) received imatinib adjuvant therapy. The starting dose of imatinib was 400 mg/day for 314 patients and 300 mg/day for 77 patients. The other 102 patients did not undergo adjuvant therapy due to a fear of adverse events due to imatinib (*N* = 39; 38%), economic reasons (26; 26%), advanced age (20; 20%), patient refusal for unidentifiable reasons (20; 20%), and comorbidities (15; 15%), as shown in Table 2. Some patients and investigators doubted the evidence of improvements in overall survival (OS) due to adjuvant therapy and were concerned about the potential induction of drug resistance by the therapy. Patients without adjuvant therapy were older, exhibited poorer performance statuses (PSs), and more frequently had nongastric GISTs (Table 3). The GISTs of patients with adjuvant therapy were larger, showed a higher mitotic count, and ruptured more frequently than those of patients without adjuvant therapy. Multivariate analysis indicated that age (HR 0.71; 95% CI 0.58–0.88; *P* = 0.002), tumor size (HR for > 10 cm vs < 5 cm, 3.87; 95% CI 1.72–8.74; *P* = 0.001), mitotic count (HR for > 10/50 HPFs vs < 5/50, 3.54; 95% CI 1.84–6.79; *P* < 0.001), tumor rupture (HR 3.69; 95% CI 1.43–9.52; *P* = 0.007) and PS (HR 0.55; 95% CI 0.31–0.99; *P* = 0.046) were independent factors of the administration of adjuvant therapy (Supplemental Table 2).

**Table 2:** Reasons for no adjuvant therapy

Reasons for no adjuvant therapy	Total *N* = 102 (%)
Fear of adverse events of imatinib	39 (38.2%)
Economic reasons	26 (25.5%)
Advanced age	20 (19.6%)
Patient refusal unknown	20 (19.6%)
Comorbidities	15 (14.7%)
Doubts for evidences of OS	10 (9.8%)
Re-review results of central pathology	3 (2.9%)
Fear of resistant mutations	2 (2.0%)
Poor PS	1 (1.0%)
Others	2 (2.0%)

**Table 3:** Clinicopathological features of adjuvant and non-adjuvant patients

Total	Non-adjuvant (*N* = 102)	Adjuvant (*N* = 432)	*P* value
Age (median, IQR; years)	70.5 (60–80)	64 (55–71)	< 0.001
Gender			
Male	58 (56.9%)	236 (54.6%)	0.766
Female	44 (43.1%)	196 (45.4%)	
Location			
Esophagus	5 (4.9%)	2 (0.5%)	0.005
Stomach	56 (54.9%)	262 (60.6%)	
Small intestine	30 (29.4%)	133 (30.8%)	
Colon and rectum	9 (8.8%)	23 (5.3%)	
Others	2 (2.0%)	12 (2.8%)	
PS			
0	77 (75.5%)	370 (85.6%)	0.004
1	20 (19.6%)	56 (13.0%)	
2	3 (2.9%)	1 (0.2%)	
3	2 (2.0%)	1 (0.2%)	
Neoadjuvant			
(−)	95 (93.1%)	380 (88.0%)	0.186
(+)	7 (6.9%)	52 (12.0%)	
Surgery			
Open	71 (69.6%)	316 (73.1%)	0.551
Laparoscopic	31 (30.4%)	116 (26.9%)	
Curability of surgery			
R0	100 (98.0%)	417 (96.5%)	0.639
R1	2 (2.0%)	15 (3.5%)	
Tumor size (median, IQR: cm)			
	6.5 (5.1–10.0)	7.7 (5.5–12.0)	0.035
Unavailable	0	1	
Mitosis (median, IQR:/50HPF) at local			
	8 (4–17)	11 (5–25)	0.009
Unavailable	6	31	
Tumor rupture			
No	94 (92.2%)	365 (84.5%)	0.042
Yes	6 (5.9%)	60 (13.9%)	
Unavailable	2 (2.0%)	7 (1.6%)	
Histological types			
Spindle	86 (84.3%)	360 (83.3%)	0.343
Epithelioid	1 (1.0%)	16 (3.7%)	
Mixed	10 (9.8%)	36 (8.3%)	
Unavailable	5 (4.9%)	20 (4.6%)	
Genotyping			
KIT	84 (82.4%)	372 (86.1%)	0.829
PDGFRA	4 (3.9%)	14 (3.2%)	
Wild type	8 (7.8%)	29 (6.7%)	
Unavailable	10 (9.8%)	31 (7.2%)	

Next, we evaluated pathological concordance for high-risk GISTs between local, general pathologists and a central GIST specialist and surveyed the influence of the results of the central pathological examination on treatment changes. Of 534 GISTs that had been locally diagnosed as high-risk GISTs, 19 tumors (3.6%) were diagnosed as non-GISTs in central pathology (Table 4; the pathological features of which are summarized in Supplemental Table 3). The non-GISTs showed unusual locations, including extragastrointestinal locations, negative staining for KIT and DOG1 in the central pathological diagnosis, and no mutations in the *KIT* and *PDGFRA* genes (Supplemental Tables 3 and 4). Of 515 centrally certified GISTs, 93 (18.1%) GISTs were reclassified into lower categories due to different mitotic counts in the central pathological diagnosis (Table 4). While the local and central mitotic counts were well correlated, the former were significantly higher than the latter (Supplemental Fig. 2).

**Table 4:** Concordance between local and central diagnosis

Central pathology	Pts no. (*N* = 534)	%
Histology		
Non-GIST	19	3.6
GIST	515	96.4
Risk re-classification of true GISTs		
Risk classification in the central pathology (*N* = 515)		
High risk	411	79.8
Intermediate	64	12.4
Low	25	4.9
Very low	4	0.8

Since the guidelines do not recommend imatinib adjuvant therapy for non-GISTs or for GISTs with *PDGFRA* D842V mutations, which are considered to be resistant to all available tyrosine kinase inhibitors, including imatinib, we used the questionnaire to examine changes in the clinical practice of local hospitals after receiving the central pathology results (Table 5). Of 19 non-GIST patients in the central pathological diagnosis, 10 patients who were undergoing imatinib adjuvant therapy at the time the results were returned were evaluable. Six patients (60%) stopped imatinib adjuvant therapy, and the other 4 continued the therapy despite being diagnosed as having a non-GIST by central pathology. Similarly, 8 patients with GISTs with *PDGFRA* D842V mutations were evaluable; 5 (63%) patients stopped adjuvant therapy after the central pathology results were reported, and the other three continued. Central pathological review and genetic analysis results were shared with all patients who have had treatment changes to discontinue adjuvant therapy, however, central reports were not always shared in the other cases. Some of the latter patients had already stopped adjuvant therapy before central reporting due to disease relapses or adverse events. Among patients with wild-type GISTs, all 7 evaluable patients continued imatinib adjuvant therapy after the reporting. However, almost all investigators participating in this study answered that they always refer to the GIST guidelines in their clinical practice and that they treat GIST patients according to the guidelines (Supplemental Table 3).

**Table 5:** Changes in adjuvant therapy after central pathology

	Non-GIST (*N* = 19)	PDFGRA exon18 D842V (*N* = 17)	Wild type (*N* = 19)
Initially no adjuvant therapy	5	3	4
No. of patients received adjuvant	14	14	15
Unevaluable due to other reasons^a^	4	4	8
No. of evaluable patients with adjuvant	10	8	7
Stopped by central pathology	6 (60%)	5 (63%)	0 (0%)
Continued after central pathology	4 (40%)	3 (38%)	7 (100%)

^a^Imatinib adjuvant therapy was already stopped before returning central pathology due to relapses or patients’ refusal of imatinib due to adverse events

## Discussion

Diagnosis and treatment according to the clinical practice guidelines and specialized multidisciplinary management have been indicated to be associated with improved QoL and survival [1, 10, 16], especially in rare cancers. Among GISTs, high-risk GISTs are considered to require a multidisciplinary approach, such as adjuvant therapy, to improve the prognostic outcome [6-8]. In this study, we examined the adherence to the guidelines and the concordance of the pathological diagnosis in the real world using data obtained from a prospective registry of high-risk GISTs with central pathology. Two-thirds of participating hospitals reported, on average, annual experiences of less than 10 primary GISTs/year (Supplemental Fig. 3), suggesting that the study may include standard acute care hospitals in Japan. The study indicated that adjuvant therapy was performed for 81% of high-risk GIST patients diagnosed locally. This value appears to be slightly better than those in previous reports from the USA and Canada [11-13, 17], in which adjuvant therapy was performed for 68% of high-risk GIST patients and for nearly 70% in high-volume centers. The difference may be due to the different timeframe of our study (2012–2015) and the American studies (2004–2009 for Pisters [11], 2006–2007 for Bilimoria [12], or 2009–2012 for Bischof [13]). In fact, the proportion of patients receiving adjuvant therapy has increased over time. Bilimoria et al. [12] indicated that adjuvant therapy was more likely to be administered to a patient with a large tumor ( > 6 cm), a high-grade tumor, and a positive marginal status at a high-volume cancer center in a recent time period. Bischof et al. [17] indicated that the tumor size, mitotic rate, and neoadjuvant therapy were independent determinants of adjuvant therapy after adjusting for confounding variables. In our study, the tumor size, mitotic rate, and tumor rupture were found to be positive selection factors for adjuvant therapy, while age and PS were negative selection factors. The reported reasons for not undergoing adjuvant therapy are reasonable, and taken together, these findings indicate that treatment options are generally shared and selected with patients according to their conditions and intention in the real world.

Previously, pathological diagnosis has been suggested to not always be consistent among pathologists, even in clinical trials [2, 18]. The inconsistency among pathologists is greater for rare cancers. For sarcomas, several retrospective studies have found a major discordance rate of more than 10% between sarcoma pathologists and general pathologists [3−5], which may result in delayed diagnosis and poor patient outcomes [1]. Contrary to soft tissue sarcomas, GISTs may not show such a high major discordance rate; for GISTs, the diagnostic criteria are indicated by the guidelines [6-8, 19]. Our major discordance rate for high-risk GISTs (3.6%) appears to be better than that in European reports, in which pathological concordance according to the tumor category is 90.6% and 87.0% for GISTs and soft tissue sarcomas, respectively [5, 20]. Our study indicates that key factors of concordance may include positive KIT and DOG1 staining and genotyping and that special care may be required for extragastrointestinal GISTs. In this study, we observed a minor discordance rate of 17.4% in the risk classification, which was caused by different mitotic rates between local and central pathologists. Thus, in total, nearly 20% of discordance may exist between general and disease-specific pathologists. The local and central mitotic counts of pathologists were well correlated; however, the former were significantly higher than the latter. This may be caused by differences in the microscopic fields and sections between the two pathologists, the field number of each microscope, and mitotic figure counts. The field number of the central microscope was one of the widest, as mentioned in the “Patients and methods” section. Most sections submitted for central pathology likely originate from areas similar to those used for local diagnosis, and mitotic differences caused by differences in the paraffin sections may disappear after the accumulation of sufficient numbers. Thus, different mitotic counts between pathologists may be speculated to be causative; however, we did not have a central review of GISTs other than high-risk tumors in this study, so we could not identify the true causes of the differences between the local and central mitotic counts.

Practical discordance in the pathological diagnosis of rare cancers may be resolved by central pathology or pathological consultation [18]. No reports, however, have tracked and evaluated the treatment changes in local hospitals after the return of the central pathology report, including the genotype. We found that for both patients with non-GISTs and GISTs with the *PDGFRA* D842V mutation, 40% of patients continued imatinib adjuvant therapy, although the guidelines do not recommend the therapy and local investigators themselves reported that their clinical practices comply with the guidelines, suggesting that central pathology results may not always be used locally. Interestingly, adjuvant therapy was continued in wild-type GIST after reporting central pathology as the guidelines suggest. This study examined a limited number of Japanese patients; however, it may be speculated that similar phenomena may occur in western countries and other Asian countries. In fact, discordance in pathological diagnosis appears to be universal in rare cancer as mentioned above. Taken together, the results suggest that in addition to central pathology, other methods might be required to facilitate treatment changes in local hospitals.

The study has several limitations. As adherence to the guidelines was high and the pathological discordance was small in our series, the power of the statistical analysis may not be sufficient to identify factors contributing to pathological discordance or guideline adherence. We only examined clinical practice in Japan. These phenomena should be evaluated in an international large-scale study with real-world data. Our study is based on a prospective registry involving general hospitals in Japan, and the data are nearly complete, with a high follow-up rate. Since this study used the fixed baseline data of the registry study, we cannot evaluate the effects of treatment adherence, adjuvant therapy completion rate or prognostic outcome of patients with high-risk GISTs with or without adjuvant therapy, which may require at least another 2 years.

In summary, we have been maintaining a prospective registry of high-risk GISTs and analyzed the baseline data along with the results of a questionnaire survey administered to participating investigators. In the registry study, adjuvant therapy was administered to 81% of high-risk GIST patients in the real world. The reasons some patients did not undergo adjuvant therapy were a fear of adverse events, economic reasons, advanced age and comorbidities. In the study, the rates of major and minor discordance in the pathological diagnosis of GISTs were 3.6% and 17.4%, respectively, and these values are better than those observed for soft tissue sarcomas. Although central pathology and genotyping may fill the gap between general and specialized pathology, further improvements and other methods may be required to improve clinical practice in general hospitals.

## Electronic supplementary material

Below is the link to the electronic supplementary material.

Supplementary file1 (PDF 116 kb)

Supplementary file2 (PDF 146 kb)

## Abbreviations

GIST: Gastrointestinal stromal tumor
QOL: Quality of life
NCCN: National Comprehensive Cancer Network
ESMO: European Society for Medical Oncology
CI: Confidence interval
PDGFRA: Platelet-derived growth factor receptor alpha
NIH: National Institutes of Health
IRB: Institutional review board
IHC: Immunohistochemistry
HPF: High-power field
PS: Performance status
