# Supplementary material for: Adherence to the guidelines and the pathological diagnosis of high-risk gastrointestinal stromal tumors in the real world
Source: Gastric Cancer. 2019 Apr 30;23(1):118–25. doi: 10.1007/s10120-019-00966-4 (PMC6942594; doi:10.1007/s10120-019-00966-4)

Supplement Figure 1. The scheme of the registry study and consort diagram

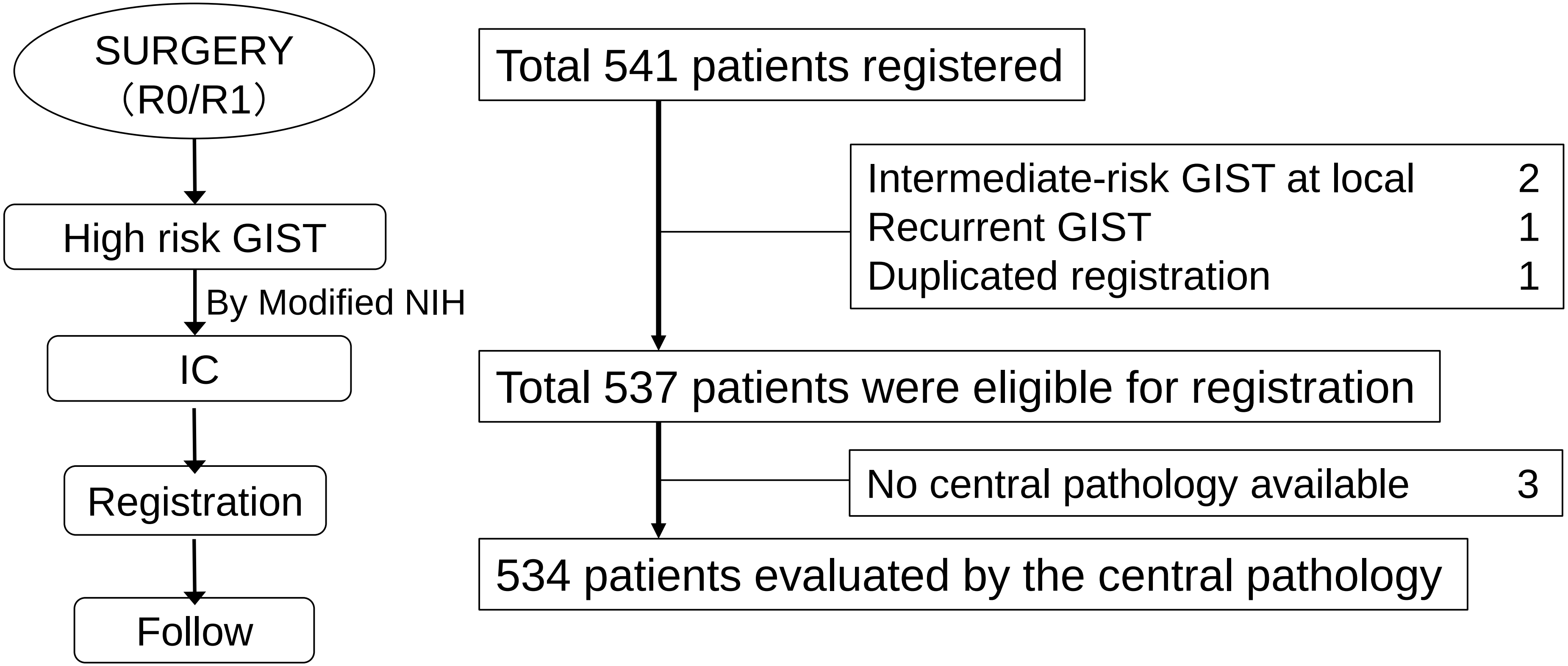

Supplement Figure 2. Concordance in the mitotic count between local and central pathology

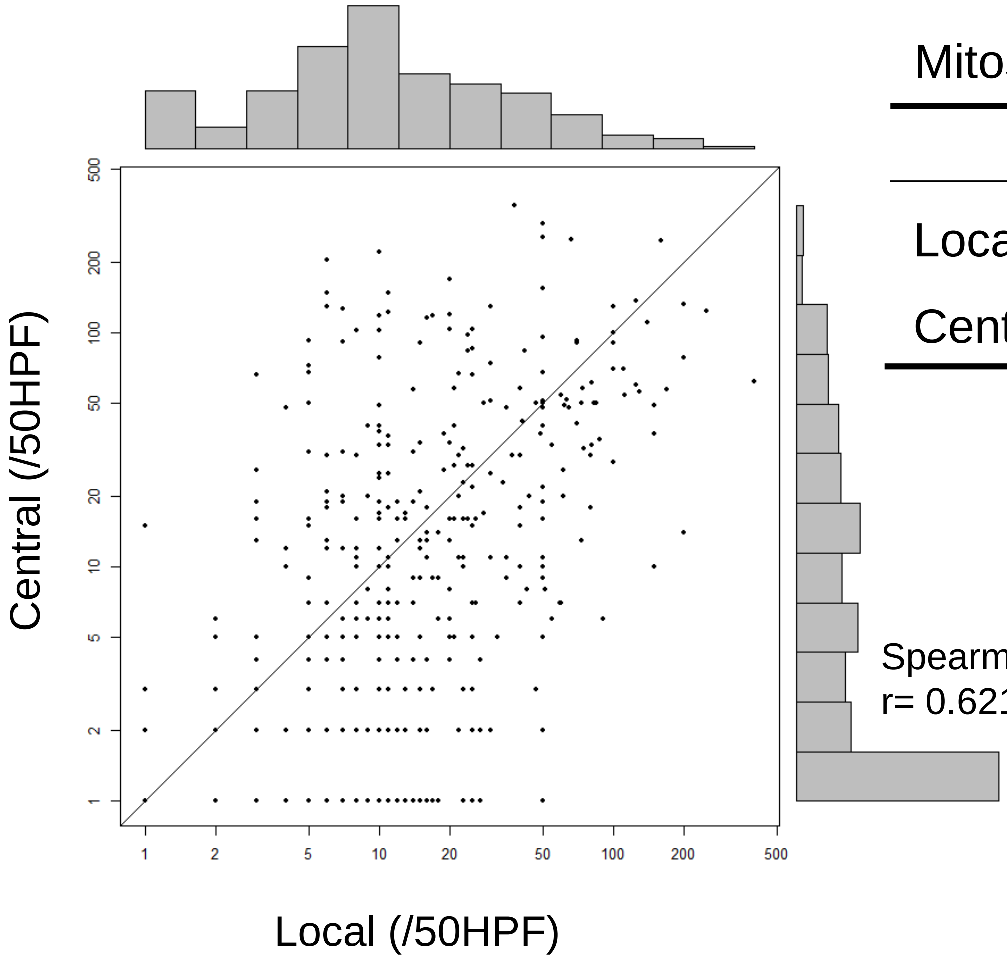

Mitosis (/50HPF)

	Median (IQR)	N
Local	10 (23 – 5)	494
Central	5 (23 – 1)	494

Signed rank test: P<0.001
IQR: interquartile range

Spearman's correlation coefficient
r= 0.621 (p<0.001)

Supplement Figure 3. Questionary survey to each hospital and investigator

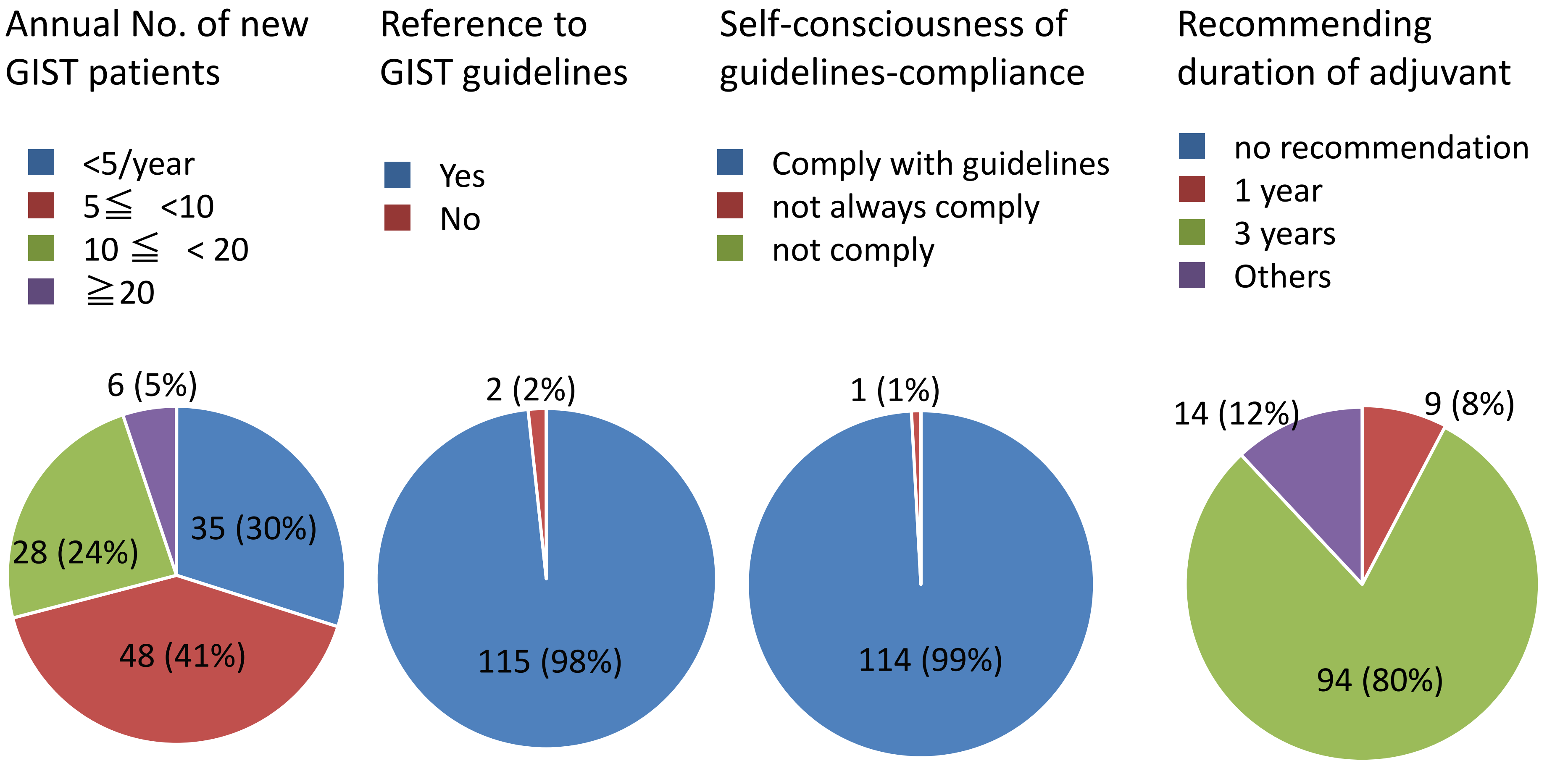

Supplement: Supplementary file 1 — Supplementary file1 (PDF 116 kb) [file 10120_2019_966_MOESM1_ESM.pdf]
