# Supplementary material for: Adherence to the guidelines and the pathological diagnosis of high-risk gastrointestinal stromal tumors in the real world
Source: Gastric Cancer. 2019 Apr 30;23(1):118–25. doi: 10.1007/s10120-019-00966-4 (PMC6942594; doi:10.1007/s10120-019-00966-4)
Supplement: Supplementary file 2 — Supplementary file2 (PDF 146 kb) [file 10120_2019_966_MOESM2_ESM.pdf]

Supplement Table 1. Surgical Morbidity

Post-operative Morbidity		No. of patients
Any morbidity		63
SSI	Intraabdominal	18
	Superficial	17
Ileus	Paralytic	14
	Mechanical	8
Pneumonia		6
Ascites		4
Bleeding		4

Supplement Table 2. Logistic regression analysis for adjuvant therapy

		Odds Ratio	95% CI	p value
Age (years)	per 10 years	0.71	0.58 - 0.88	0.002
Tumor size (cm)	5-10cm *vs* <5cm	1.48	0.78 - 2.84	0.255
	>10cm *vs* <5cm	3.87	1.72 - 8.74	0.001
Mitosis (/50HPF)	6-10 *vs* <5	1.54	0.82 - 2.88	0.456
	>10 *vs* <5	3.54	1.84 - 6.79	0.000
Tumor rupture	Presence *vs* Absence	3.69	1.43 - 9.52	0.007
PS	1-or-more vs 0	0.55	0.31 - 0.99	0.046

Supplement Table 3. Cases of complete discordance between local and central pathology

Case	Dx	Origin	Tumor size (cm)	Mitosis	KIT	DOG1	CD34	Desmin	KIT・PDGFRA mutation	Comments
1	Local<br>Central	peritoneum	7.8	9<br>20	positive<br>+/-	 negative	positive<br>+/-		None	Sarcoma, NOS: KIT(-), DOG1(-)
2	Local<br>Central	small intestine	9.0	5<br>68	positive<br>negative	 negative	+/-<br>negative	negative 	n.e.	Sarcoma, NOS: KIT(-), DOG1(-)
3	Local<br>Central	small intestine	15.0	20<br>104	 negative	 negative	 negative		None	Sarcoma, NOS: KIT(-), DOG1(-)
4	Local<br>Central	stomach	34.0	10<br>19	positive<br>negative	 negative	 +/-		None	Leiomyosarcoma suspected: Desmin (+), SMA (+/-)
5	Local<br>Central	stomach	6.5	10<br>1	positive<br>negative	 negative	positive<br>positive	negative 	None	Leiomyosarcoma suspected: Desmin (-), SMA (+/-)
6	Local<br>Central	small intestine	12.8	0<br>0	positive<br>negative	 negative	negative<br>negative		None	Desmoid: nuclear β-catenin (+)
7	Local<br>Central	larynx	4.0	50<br>1	positive<br>negative	 +/-	positive<br>negative	positive 	None	compatible with Leiomyosarcoma: SMA(+), Desmin (+)
8	Local<br>Central	esophagus	7.2	5<br>0	positive<br>negative	 negative	positive<br>negative	positive 	None	Leiomyoma: KIT (-), DOG1 (-)
9	Local<br>Central	colon	15.0	0<br>0	+/-<br>negative	 negative	negative<br>negative		None	Desmoid: β-catenin exon 3 Thr41Ala
10	Local<br>Central	peritoneum	8.0	5<br>72	positive<br>negative	 negative	negative<br>negative	negative 	None	Sarcoma, NOS: KIT(-), DOG1(-)
11	Local<br>Central	small intestine	8.0	1<br>0	+/-<br>negative	 negative	positive<br>negative		None	Desmoid: nuclear β-catenin+, β-catenin exon 3 Thr41Ala
12	Local<br>Central	stomach	2.8	18<br>0	positive<br>negative	 negative	positive<br>+/-	positive 	None	Leiomyosarcoma suspected: Desmin (+), SMA(+)
13	Local<br>Central	colon	13.5	42<br>84	positive<br>+/-	 +/-	negative<br>negative		None	Sarcoma, NOS: KIT(-), DOG1(-)
14	Local<br>Central	omentum	10.0	7<br>1	positive<br>negative	 negative	positive<br>positive		None	Solitary fibrous tumor NAB2-STAT61 fusion gene (+),
15	Local<br>Central	mesenterium	5.5	200<br>14	positive<br>negative	 negative	positive<br>+/-	positive 	None	Leiomyosarcoma suspected: Desmin (+) in local hospital
16	Local<br>Central	stomach	9.5	23<br>1	+/-<br>negative	 negative	positive<br>negative	negative 	None	Sarcoma, NOS: KIT(-), DOG1(-)
17	Local<br>Central	stomach	6.0	10<br>118	negative<br>negative	 negative	positive<br>negative	negative 	None	Sarcoma, NOS: KIT(-), DOG1(-)
18	Local<br>Central	small intestine	14.0	2<br>0	positive<br>negative	 negative	negative<br>negative	negative 	None	Desmoid: β-catenin exon 3 Thr41Ala
19	Local<br>Central	mesenterium	4.5	81<br>33	positive<br>negative	 negative	negative<br>negative		None	Leiomyosarcoma: α-SMA (+), Desmin (+)

Common features of non-GISTs in the central pathology are:KIT-negative & DOG1-negative and no mutations in KIT and PDGFRA, although they were diagnosed KIT-positive or –weaklypositive, and/or CD34-positive.

Supplement Table 4. Comparison of clinicopathological features between GIST and non-GIST

Total		GIST (N=515)	Non-GIST (N=19)	P value
Age (median, IQR; years)		65 (56-72)	60 (40-72)	0.076
Gender	Male	280 (54.4%)	14 (73.7%)	0.154
	Female	235 (45.6%)	5 (26.3%)	
Location	Esophagus	6 (1.2%)	1 (5.3%)	<0.0001
	Stomach	313 (60.8%)	5 (26.3%)	
	Small intestine	158 (30.7%)	5 (26.3%)	
	Colon & rectum	30 (5.8%)	2 (10.5%)	
	Others	8 (1.6%)	6 (31.6%)	
Neoadjuvant	(-)	456 (88.5%)	19 (100%)	0.233
	(+)	59 (11.5%)	0 (0%)	
Surgery	Open	373 (72.4%)	14 (73.7%)	1.000
	Laparoscopic	142 (27.6%)	5 (26.3%)	
Curability of surgery	R0	498 (96.7%)	19 (100%)	0.889
	R1	17 (3.3%)	0 (0%)	
Tumor size (median, IQR: cm)		7.5 (5.5 – 11.3)	8.0 (6.0 – 13.5)	0.343
	unavailable	1	0	
Mitosis (median, IQR: /50HPF) at local		10 (5 – 23)	10 (5 – 23)	0.666
	unavailable	38	0	
Tumor rupture	No	442 (85.8%)	17 (89.5%)	1.000
	Yes	64 (12.4%)	2 (10.5%)	
	Unknown	9 (1.7%)	0 (0%)	
Histological types	Spindle	433 (84.1%)	13 (68.4%)	0.113
	Epithelioid	15 (2.9%)	2 (10.5%)	
	Mixed	45 (8.7%)	1 (5.3%)	
	unavailable	22 (4.3%)	3 (15.8%)	
KIT in central pathology	(+)	499 (96.9)	0 (0)	<0.0001
	(±)	12 (2.3)	2 (10.5)	
	(-)	3 (0.6)	17 (89.5)	
	unavailable	1 (0.2)	0 (0)	
DOG1 in central pathology	(+)	501 (97.3)	0 (0)	<0.0001
	(±)	11 (2.1)	2 (10.5)	
	(-)	2 (0.4)	17 (89.5)	
	unavailable	1 (0.2)	0 (0)	
Genotyping	KIT	456 (88.5%)	0 (0%)	<0.0001
	PDGFRA	18 (3.5%)	0 (0%)	
	Wild type	19 (3.7%)	18 (94.7%)	
	unavailable	22 (4.3%)	1 (5.3%)	

Supplement Table 5. The members of the STAR ReGISTry Study Group

Following Institutions and Hospitals, and Investigators in the parentheses are the members of the STAR ReGISTry Study Group;

Osaka Police Hospital (Takuro Saito), Hokkaido University Hospital (Yoshito Komatsu), Kobe City Medical Center General Hospital (Masato Kondo), Kanagawa Cancer Center (Tsutomu Hayashi), Kyoto University Hospital (Yoshiharu Sakai), National Cancer Center Hospital East (Naoto Gotoda), Chiba Cancer Center Hospital (Nobuhiro Takiguchi), Hyogo Prefectural Amagasaki General Medical Center (Atsuhiko Maki), Kumamoto University Hospital (Hideo Baba), Juntendo University Shizuoka Hospital (Hajime Orita), Niigata Cancer Center Hospital (Hiroshi Yabusaki), Yokohama Rosai Hospital (Gaku Chiguchi, Kyoto Katsura Hospital (Dai Manaka), Tokai University Hospital (Kazuhito Nabeshima), Asahikawa-Kosei General Hospital (Hiromitsu Akabane), Obihiro-Kosei General Hospital (Koichi Ono), Keio University Hospital (Norihito Wada), Toyama Prefectural Central Hospital (Masahide Kaji), Gifu University Hospital (Kazuhiro Yoshida, Matsuyama Red Cross Hospital (Ikuo Takahashi), Osaka General Medical Center (Kazumasa Fujitani), Nara Medical University Hospital (Sohei Matsumoto), Osaka City General Hospital (Yutaka Tamamori), Tottori University Hospital (Hiroaki Saito), Kitano Hospital The Tazuke Kofukai Medical Research Institute (Shugo Ueda), Kawasaki Medical School Hospital (Masahiro Yamamura, Japanese Red Cross Kumamoto Hospital (Eiji Tanaka), Jichi Medical University Hospital (Hirofumi Fujii), Yamaguchi University Hospital (Shigefumi Yoshino), St. Luke's International Hospital (Akihiro Suzuki), University Hospital Kyoto Prefectural University of Medicine (Eigo Otsuji), Tokyo Medical University Hachioji Medical Center (Shigeyuki Kawachi), Osaka University Hospital (Tsuyoshi Takahashi, Osaka City University Hospital (Kazuya Muguruma, Niigata University Medical and Dental Hospital (Suguru Ishikawa), Sanda City Hospital (Masaaki Mitsutsuji), Saiseikai Kumamoto Hospital (Hiroshi Takamori), Kimitsu Chuo Hospital (Takashi Kaiho), Hitachi General Hospital (Akihiro Sako), Aichi Cancer Center Hospital (Seiji Ito), National Hospital Organization Chiba Medical Center (Masahiro Mori), Center Hospital of the National Center for Global Health and Medicine (Makoto Tokuhara), University of Yamanashi Hospital (Yoshihiko Kawaguchi), Hiroshima City Asa Citizens Hospital (Naoki Hirabayashi), Ehime University Hospital (Motohira Yoshida), Yokohama

Municipal Citizen's Hospital (Masazumi Takahashi), Hyogo Prefectural Kakogawa Medical Center (Shiro Takase), Kitasato University East Hospital (Keishi Yamashita), Tokyo Metropolitan Cancer and Infectious Diseases Center Komagome Hospital (Yoshiaki Iwasaki), Kizawa Memorial Hospital (Yutaka Ozeki), Keiyukai Sapporo Hospital (Yasunori Nishida), Sakai City Medical Center Yutaka Kimura), Iwate Medical University (Keisuke Koeda), Kaizuka City Hospital (Toshimasa Tsujinaka), Japanese Red Cross Nagoya Daini Hospital (Hiroshi Kanie), National Hospital Organization Shikoku Cancer Center (Shinji Hato), Seichokai Fuchu Hospital (Junya Morimoto), Sendai City Medical Center (Hiroshi Honda), National Hospital Organization Kure Medical Center and Chugoku Cancer Center (Hirotaka Tashiro), Kobe University Hospital (Yoshihiro Kakeji), National Hospital Organization Kyoto Medical Center (Hiroaki Hata), Toyama University Hospital (Toshiro Sugiyama), Sapporo Medical University Hospital (Takayuki Nobuoka), Teikyo University Hospital (Ryoji Fukushima), South Miyagi Medical Center (Katsuro Sugiyama), Osaka Rosai Hospital (Junichi Hasegawa), Tenri Hospital (Tsunehiro Yoshimura), National Cancer Center Hospital (Atsuo Takashima), Yokohama City University Medical Center (Chikara Kunisaki), Saiseikai Utsunomiya Hospital (Hiroharu Shinozaki), Steel Memorial Muroran Hospital (Naoto Senmaru), Toyonaka Municipal Hospital (Hiroshi Imamura), Oita University Hospital (Satoshi Otsu), Nagoya University Hospital (Daisuke Kobayashi), Matsushita Memorial Hospital (Akinori Noguchi), Hakodate Goryoukaku Hospital (Akinori Takagane), Tokyo Women's Medical University Yachiyo Medical Center (Atsushi Mitsunaga), Kansai Rosai Hospital (Shigeyuki Tamura), Yao Municipal Hospital (Jin Matsuyama), Nishinomiya Municipal Central Hospital (Yoshio Oka, Iizuka Hospital (Kiyoshi Kajiyama), Tokyo Women's Medical University Hospital (Takuji Yamada), Tokyo Medical University Hospital (Sumito Hoshino), Hiroshima University Hospital (Hideki Ohdan), National Hospital Organization Okayama Medical Center (Tomokazu Kakishita), The Jikei University School of Medicine Hospital (Katsuhiko Yanaga), Yokohama City University Hospital (Yasushi Rino), Japanese Red Cross Ashikaga Hospital (Takayuki Takahashi), Chiba University Hospital (Hisahiro Matsubara), Okayama Rosai Hospital (Masahiro Ishizaki), Kansai Medical University (Songtae Kim), Ishikawa Prefectural Central Hospital (Noriyuki Inaki), Shimane University Hospital (Noriyuki Hirahara), National Hospital Organization Kyushu Cancer Center (Masaru Morita), Sanjo General

Hospital (Tatsuo Kanda), Kawasaki Medical School Hospital (Tomoki Yamatsuji, Hoshigaoka Medical Center (Mitsutoshi Tatsumi), Suita Municipal Hospital (Chikara Ebisui), International University of Health and Welfare, Mita Hospital (Yoshifumi Ikeda), Iwate Prefectural Central Hospital (Tsukasa Inoue), Yamagata University Hospital (Wataru Kimura), Otemae Hospital (Hiroyuki Nakaba), Saiseikai Nara Hospital (Takamune Shibaji), National Hospital Organization Osaka Minami Medical Center (Taichi Tatsubayashi), Nagano Municipal Hospital (Masahiro Sakon), National Hospital Organization Tokyo Medical Center (Yo Isobe), Tokushima University Hospital (Mitsuo Shimada), Hyogo College of Medicine College Hospital (Mitsuru Sasako), Naha City Hospital (Hirofumi Tomori), Rinku General Medical Center (Koichi Demura), Kawasaki Hospital (Masahiro Fujikawa), National Hospital Organization Saitama National Hospital (Hirohito Ishizuka), Kashiwara Municipal Hospital (Masanari Tendo), Saitama Medical University International Medical Center (Shinichi Sakuramoto), Shimane Prefectural Central Hospital (Akiyoshi Kanazawa), Yamagata Prefectural Central Hospital (Norimasa Fukushima), Saga-ken Medical Center Koseikan (Seiji Sato), Okayama Saiseikai General Hospital (Takaomi Takahata), Clinical Research Institute National Hospital Organization Kyushu Medical Center (Tetsuya Kusumoto), Osaka International Cancer Institute (Takeshi Omori), Tohoku University Hospital (Masanobu Takahashi), Akita University Hospital (Masahiro Inoue), Kagawa Prefectural Central Hospital (Norimitsu Tanaka), Hiroshima City Hiroshima Citizens Hospital (Motoki Ninomiya), Hikone Municipal Hospital (Yasufumi Teramura)
